# The Use of Behavioral Modalities for Headache During Pregnancy and Breastfeeding

**DOI:** 10.1007/s11916-021-00980-1

**Published:** 2021-10-19

**Authors:** Nina Riggins, Annika Ehrlich

**Affiliations:** 1 UCSF Medical School, UCSF Headache Center, University of California, San Francisco, CA USA; 2 UCSF Headache Center, UCSF School of Nursing, University of California, San Francisco, CA USA

**Keywords:** Headache, Migraine, Pregnancy, Lactation, Cognitive behavioral therapy, Meditation, Relaxation

## Abstract

**Purpose of Review:**

Migraine is primary headache which commonly affects women of childbearing age. Migraine and other primary headache disorders are also common during pregnancy. Understanding which treatments are effective and can be safely given to patients with primary headache during pregnancy and lactation is essential in supporting these patients before, during, and after childbirth. Behavioral modalities have the potential to improve the health of both mother and baby, while empowering patients to make informed decisions in family planning and creating future treatment plans.

**Recent Findings:**

Research shows that behavioral therapies can be powerful tools to treat pain conditions with minimal side effects. Recent literature prioritizes behavioral therapies in preparation for pregnancy, during pregnancy, and during lactation due to the superior safety profile of such therapies. Digital resources for behavioral therapy are another well-received recent direction supported by growing evidence of both efficacy and safety. Popular with patients and headache specialists, digital behavioral therapy has taken various forms during the pandemic, such as telemedicine, online psychology support groups, and smartphone applications that patients can interact with on their own time.

**Summary:**

In summary, the purpose of this review is to equip providers with important information and updates on the use of behavioral modalities for the treatment of primary headache during pregnancy and lactation.

## Introduction

Migraine and other primary headache disorders (such as trigeminal autonomic cephalalgias) are common during pregnancy affecting 10–17% of pregnancies [1••].

Recent research suggests that, despite the possible improvement of a majority of patients with migraine by the third trimester, one in five women avoided pregnancy because of migraine with concerns that migraine will get worse, affect the health of their child, or both [1••, 2,3,4]. It is also common for women undergoing headache treatment to express concern before pregnancy about having to stop such treatments that were effective because they are not approved safe during pregnancy. An example of such a situation is a woman who has improvement of migraine while using calcitonin gene-related peptide (CGRP) antibodies for migraine prevention and who is taken off this medication months prior to becoming pregnant due to lack of evidence that it is safe during pregnancy. The crucial question becomes, what are safe and effective treatments for migraine in preparation for pregnancy, during pregnancy and during lactation?

Stress and hormonal changes are known triggers for migraine attacks [5]. There is a dual challenge to give a woman the most effective treatment that is safe for both her and her baby. There is a realization that undertreatment of headache during pregnancy and lactation can lead to negative sequences for women and for familial relations, such as interfering with mother–child bonding [1••].

Challenges with risks and benefits of medications, procedures, and devices make behavioral modalities an attractive option for management of headache during pregnancy and lactation, and in preparation for pregnancy. In this review, we identify potential behavioral interventions to consider before and during pregnancy and after childbirth.

Because literature on this subject is scarce, we used direct evidence—such as research discussing effects and side effects during pregnancy and lactation—and also indirect evidence—behavioral modalities we have and future directions of research in the general population of people living with primary headache.

Therefore, the goal of this review is to equip providers with important information and updates regarding the use of behavioral modalities for the safe and effective treatment of primary headache during pregnancy and lactation.

### Research Methods

We consulted with a research librarian affiliated with our institution, who assisted with our PubMed search regarding any published articles with the following keywords/phrases:

*(pregnancy[ti] OR pregnant[ti] OR lactation[ti] OR lactating[ti] OR breast feeding[ti] OR breastfeeding[ti] OR pregnancy[ot] OR pregnant[ot] OR lactation[ot] OR lactating[ot] OR breast feeding[ot] OR breastfeeding[ot] OR pregnancy[mh] OR lactation[mh] OR breast feeding[mh) AND (primary headache OR "headache disorders, primary" [mh] OR migraine OR cluster headache) AND (behavioral therapy OR exercise OR yoga OR meditation OR mindfulness OR stress reduction)*

With these specific search terms, 150 articles were found with no time limitations. We prioritized the most recent evidence (within the past 3 years) regarding behavioral therapies specifically for treatment of headache in pregnancy and breastfeeding and included 48 articles in this review.

### Disclosure of Possible Bias and Research Obstacles

Before we begin the substantive portion of the review, we must disclose possible bias and research obstacles.

Psychiatric comorbidities such as depression and anxiety can be seen more frequently in patients with primary headache disorders [6, 7]. Behavioral modalities, such as cognitive behavioral therapy (CBT), biofeedback, progressive muscle relaxation therapy (PMR), acupuncture, and physical therapy (PT) can be beneficial for psychiatric comorbidities, and also insomnia [8]. One of the by-products of such treatment could be that migraine and other primary headaches improve as comorbidities and sleep improve. Such results have the potential to bias findings. More studies are needed to show by which exact mechanisms patients are getting better with behavioral modalities and what can be addressed the best with certain treatments.

In terms of research, there are not enough randomized clinical trials to draw conclusions based on studies in patients who are preparing for pregnancy, pregnant, or breastfeeding, and many conclusions had to be done based on general population of patients with primary headache and retrospective reviews.

Lastly, relaxation, biofeedback, and cognitive behavioral therapy are evidence‐based behavioral therapies for migraine [8, 9]. Digital resources and motivational interviewing can help patients overcome barriers to start behavioral therapy, especially during the Covid-19 pandemic [10].

### Growing Evidence of the Effectiveness of Behavioral Modalities

Behavioral modalities can help to empower patients as they learn to change body responses to pain and adapt their understanding of headache pain to a more proactive approach.

An informal survey of relevant literature and media clearly indicates that there is a growing body of evidence showing the important role of empowering patients with self-help tools and treatments for headache, including the use of a headache diary, trigger control, relaxation techniques, healthy foods and lifestyle, and regular exercise, all of which endeavor to give patients more control over their headache pain [11, 12••, 13].

Evidence accumulated over several years finds that people with primary headache disorders can achieve a 45 to 60% reduction in headache intensity and frequency with proper and regular use of biofeedback and relaxation techniques such as progressive muscle relaxation [14]. This evidence is supported by established EMG Biofeedback and the latest technology findings [14].

Technology has helped make significant improvements in understanding the mind–body connection. For example, in one study after 8 weekly CBT sessions, not only did headache frequency decrease, but structural and resting‐state blood‐oxygen‐level‐dependent contrast MRI scans showed changes in brain function and amygdala connectivity with brain regions responsible for the procession of pain, regulation of emotions, and cognitive function [15].

### Research on Headache During Pregnancy and Lactation

Based on research, lifestyle modifications are a necessary first intervention that should be discussed in preparation for pregnancy, during pregnancy, and during lactation with every patient who has primary headache [1••].

There is ample research indicating that women experience migraine more often than men. The female-to-male prevalence ratio of migraine is 3:1 [16]. Women experience a higher percentage of migraine during reproductive age [17•]. It is believed that estrogen can lower the threshold for cortical spreading depression [18].

In addition, there is a strong association between pregnancy and migraine, with studies showing 80% of women can experience migraine during pregnancy if they had it in the past [17•, 19]. Over one half of women with migraine experience recurrence of headaches within the first month after childbirth [17•, 20]. Although there is no direct evidence to suggest that breastfeeding can trigger migraine or worsen other primary headache, there is a reasonable expectation that many parents of newborns can have sleep deprivation which can trigger migraine. In this context, the lack of sleep is not due to insomnia and therefore any resulting headache may be associative.

Digital cognitive behavioral therapy is readily available for headache patients. In one proof‐of‐concept study, 34% of patients with chronic migraine who completed a digital cognitive behavioral course reverted from chronic migraine to episodic migraine [21]. In addition, 50% of the participants reported improvement in insomnia. The study provided additional evidence for the feasibility and acceptability of dCBT‐I in patients with chronic migraine and insomnia, suggesting the beneficial effects of treating both insomnia and migraine concurrently [21].

Some people may prefer in-person sessions while others may prefer smartphone-delivered therapy. The new era of headache management gives easier access and removes many of the traditional barriers to access behavioral therapy, including socioeconomic and logistical. For example, a study of 401 participants showed that patients with migraine preferred digital or in-person behavioral therapy to phone therapy [22].

### Planting the SEEDS of Successful Outcomes

The SEEDS mnemonic stands for “Sleep, Exercise, Eat, Diary, and Stress”. [11]. It has become a popular mantra among providers who care for patients living with migraine and other headache disorders. Studies show that relaxation training and biofeedback should be regularly used to assist headache prevention and help treat acute headache and migraine [17•, 23].

Behavior therapy is not limited to the USA where behavioral modifications are commonly espoused in popular media. Reports of successful behavioral therapy intervention have emerged in medical literature sampled from regions around the world. To sharpen the focus, for behavioral therapy approaches we selected biofeedback, cognitive behavioral therapy, relaxation techniques, mindfulness-based therapy, and acceptance and commitment therapy. In a review from Italy published this year (2021), the author emphasized that behavioral modalities should specifically target pregnant women due to tolerability [20]. Similar to this review, these authors selected biofeedback, cognitive behavioral therapy, relaxation techniques, mindfulness-based therapy, and acceptance and commitment therapy for their review.

Research shows that mindfulness-based therapy improves migraine and tension headache and intervene in at least one headache outcome [12••, 24]. Mindfulness and other behavioral modalities are becoming more popular while gaining more evidence of efficacy and tolerability [12••, 13, 25,26,27].

Several studies show that biofeedback is effective in the treatment of primary headaches [12••, 28]. Significantly, studies also show that behavioral therapy is not only effective when administered in conjunction with treatment using medications, but also effective on its own [29, 30•]. One formal behavioral therapy technique called Acceptance and Commitment Therapy (ACT) uses several strategies together, incorporating mindfulness, engagement in action, and behavior modification [31,32,33].

### Proven Benefits of Exercise and Mindfulness

Healthcare professionals and laypersons have long known that exercise is good for a person’s physical well-being. Over the past several years, both groups have started to recognize the mental health benefits of exercise. Most recently, there is evidence emerging that exercise has proven physical and mental health benefits in the treatment of chronic pain such as migraine and headache. While we have understood the preventative benefits of exercise, recommending it as a form of treatment is a significant step in the evolution of pain medicine. Groundbreaking research in this area clearly indicates that regular, individualized exercise-based treatments are likely to result in improvements in pain and function, including for headache and migraine [34•, 35,36,37,38].

There is emerging evidence for the beneficial role of mindfulness, yoga, and tai chi in the treatment of migraine. Mindful breathing, yoga postures, and meditation can reduce stress. Mindfulness-based interventions (MBI) have potential as a non-pharmacological treatment for migraine, primarily through the development of flexible attention capacity across sensory, cognitive, and emotional experiences [27, 30•, 34•, 39, 40].

In another study, researchers used a survey to look at outcomes of 12 weeks of yoga in females with migraine [41]. Here the researchers were interested in headache frequency, intensity, duration, and blood nitric oxide levels and functioning. Their results showed significant reduction in headache and better functioning without significant difference in the plasma levels of nitric oxide between those in yoga therapy and the control groups before and after the study.

Data indicates that yoga is a promising treatment in the management of chronic pain [42]. Researchers have worked on understanding how yoga helps chronic pain and migraine. Studies show that yoga decreases disability among people with pain, but it is not known exactly how. A validated conceptual model of how the experience of chronic pain is affected by yoga is being looked at [43], and the beneficial effects of yoga on migraine are being studied [44, 45].

Tai chi is a gentle meditative sequence of movements. It is an exercise targeted to improve health by changes in mental focus, breathing, coordination, and relaxation. There is research to show that during pregnancy women with migraine may benefit by practicing tai chi. In one study, tai chi practice was shown to help in the reduction of headache impact, improve perceptions of some aspects of physical health, and benefit mental health [46, 47].

## Conclusions

Migraine is a chronic disabling neurologic condition that can be treated with a combination of both pharmacologic and complementary and integrative health options [48]. To quote one study: “Within a biopsychosocial approach, a multidisciplinary program for headache management should be planned. It should include not only conventional pharmacological therapies but also: patient education and support, lifestyle modification (diet, physical activity, lifestyle habits, stressful conditions, etc.), and complementary measures” [12••].

Recent findings show that behavioral therapies can be powerful tools to treat pain conditions such as headache and migraine with minimal side effects. Due to the excellent safety profile of behavioral therapies, recent literature prioritizes behavioral therapies for women preparing to get pregnant, during pregnancy, and during lactation. An exciting and democratizing recent trend is digital resources for behavioral therapy, which are well received and have growing evidence for efficacy and safety. Digital behavioral therapy has greatly expanded during the Covid-19 pandemic, manifesting in different forms. Both the delivery and efficacy have been well-received by headache patients and providers. Telemedicine pain centers, online psychology support groups, and smartphone apps are ubiquitous thanks to their convenience.

Behavioral modalities may be an excellent first choice and also an option to augment other treatments for patients with headache during preparation for pregnancy, during pregnancy and lactation. Understanding which treatments are effective and can be safely offered during pregnancy and lactation to patients with primary headache is essential in supporting these patients. This has potential to improve the health of mother and baby, while giving patients the power to make informed decisions in family planning and creating lifelong treatment plans.

Lastly, there are few randomized clinical trials evaluating the efficacy and safety of behavioral modalities in patients who are pregnant and breastfeeding. Future research with emphasis on this population would be very beneficial.
